# Special Issue: “Innate Immunity to Virus Infection, 2nd Edition”

**DOI:** 10.3390/v17121616

**Published:** 2025-12-14

**Authors:** Xinyu Zhang, Caijun Sun

**Affiliations:** 1 School of Public Health (Shenzhen), Sun Yat-sen University, Shenzhen 518107, China; zhangxy569@mail2.sysu.edu.cn; 2 Shenzhen Key Laboratory of Pathogenic Microbes and Biosafety, Shenzhen Campus of Sun Yat-sen University, Shenzhen 518107, China; 3 Key Laboratory of Tropical Disease Control (Sun Yat-sen University), Ministry of Education, Guangzhou 514400, China; 4 State Key Laboratory of Anti-Infective Drug Discovery and Development, School of Pharmaceutical Sciences, Sun Yat-sen University, Guangzhou 510006, China

## 1. Introduction

The frequent emergence of highly pathogenic viruses globally has persistently threatened global health. In confronting these continuously mutating viral strains, innate immunity—humanity’s primary and most universal defense—has returned to the forefront of research. This evolutionarily conserved system serves as the first line of defense, present across diverse life forms, from bacteria and plants to humans. Unlike the highly specific but delayed adaptive immunity, innate immunity employs pattern recognition receptors (PRRs) to rapidly sensor pathogen-associated molecular patterns (PAMPs) or damage-associated molecular patterns (DAMPs). This recognition triggers immediate inflammatory responses, recruits immune cells, and activates antiviral signaling pathways within minutes [1,2]. Interferons (IFNs), key antiviral effectors of innate immunity, exert broad-spectrum antiviral activity by binding to interferon receptors (IFNAR) in an autocrine or paracrine manner [3,4]. This interaction activates the JAK-STAT signaling pathway, leading to the expression of hundreds of interferon-stimulated genes (ISGs) [5,6,7]. However, viruses have evolved numerous strategies to evade or suppress innate immune responses at various stages of infection. To investigate this complex and dynamic interplay between host innate immunity and viral infections, we launched this Special Issue (including 1st Edition and 2nd Edition) [8] to collect novel progress on innate immunity in response to viral infections. Through a multidisciplinary approach—encompassing laboratory data, bioinformatics analyses, and studies from molecular genetics to animal models and human populations—the collected works illuminate the ongoing “arms race” between viruses and their hosts, offering valuable insights for developing novel antiviral therapeutics and vaccines.

## 2. The Central Role of Interferons

Reflecting the cornerstone status of interferons in innate immunity, several articles in this Issue focus on their functions. Omara et al. [9] compared the sensitivity of HIV-1 subtype D and AD recombinant transmitted/founder viruses to type I interferon, revealing that the AD recombinant subtype exhibits greater resistance to IFN-I than subtype D. This inherent variation underscores how evolutionary pressures drive viruses to enhance transmissibility by subverting the core IFN-I defense axis. In the review on interferon’s role in chronic HIV pathogenesis, Teer et al. [10] elaborated on the dual nature of type I interferon: a crucial antiviral defender during acute infection that may transform into a driver of persistent immune activation and immunometabolic dysregulation in chronic stages, inadvertently promoting viral persistence. Spiteri et al. [11], in their study on LCMV infection, demonstrated that the absence of IFN-I signaling (via IFNAR1) removes a critical brake on the CD8+ T cell response. Subsequent blockade of the PD-L1 checkpoint pathway then results in fatal immunopathology rather than viral clearance, highlighting the indispensable role of IFN-I in coordinating adaptive immunity and preventing excessive damage. Furthermore, Chen et al. [12] uncovered a regulatory mechanism at the transcriptional level, showing that the long non-coding RNA THRIL inhibits IRF3 activation and IFN-I production, thereby dampening innate immunity and facilitating influenza virus replication. These findings provide a new molecular target for broad-spectrum antiviral strategies.

## 3. The Innate Immune Network Beyond Interferons

Beyond interferons, innate immunity comprises a broader network of effector molecules, including PRRs, ISGs, and inflammatory cytokines, which collectively mount a powerful defense. Shifting the focus upstream of IFN-I, Andrade et al. [13] investigated severe COVID-19 in children and found that single-nucleotide polymorphisms in key PRR genes (TLR3, TLR7, TLR8, and TIARP) significantly influence disease severity. This genetic evidence underscores the decisive role of innate immune recognition pathways in personalized antiviral defense. Xie et al. [14] examined the impact of c-FLIP on the fetal brain transcriptome during Zika virus infection. They observed that c-FLIP deletion alters transcriptomic profiles of innate immune-related pathways—including PRR, interferon, and inflammatory signaling—in the fetal head, and is linked to T-cell response deficits and sensory organ developmental defects. This suggests that regulators of cell survival and death, such as c-FLIP, serve as critical nodes connecting innate immunity, adaptive immunity, and viral teratogenicity. However, innate immune responses represent a double-edged sword. As highlighted in Márquez-Bandala et al.’s review [15] on influenza virus pathogenesis, a moderate innate immune response is protective, whereas excessive activation can precipitate the “cytokine storm”-mediated tissue damage, immune cell dysfunction, and, ultimately, acute respiratory distress syndrome (ARDS) and death.

## 4. From Mechanism to Clinic

In addition to fundamental mechanisms, this Special Issue also includes research extending into clinical and public health realms. Wu et al. [16] conducted a systematic review on statins and influenza susceptibility, revealing that these widely used cholesterol-lowering drugs may increase influenza risk in specific populations (e.g., women and the elderly), potentially by suppressing innate immune responses. This finding underscores the importance of considering a drug’s impact on the host’s overall immune status during clinical practice and highlights the need for enhanced disease prevention and health monitoring in such cases.

## 5. Conclusions

In summary, this Special Issue profoundly demonstrates a series of cutting-edge studies that illuminate the central role of innate immunity in antiviral defense and its complex regulatory network. Advances in understanding the nuanced regulation of interferon signaling, the influence of PRR gene polymorphisms on disease susceptibility, and the discovery of new immune regulators such as non-coding RNAs continue to deepen our insights into host–virus interactions. However, innate immunity is a double-edged sword; the timing, magnitude, and duration of its activation collectively determine the infection outcome—whether the virus is effectively cleared or immunopathological damage occurs. Future research should further elucidate viral immune evasion tactics, the links between host genetics and immune responses, and how to develop novel therapies targeting key nodes within the innate immune system. On the translational path from laboratory to clinic, we must harness the broad-spectrum defensive potential of innate immunity while remaining aware of the risks of its overactivation, ultimately paving the way for precise antiviral interventions and effective public health measures.
